# Understanding the Link between Family Caregiver Quality of Life and the Care Provided to Older People With Dementia

**DOI:** 10.1093/geroni/igaa057.500

**Published:** 2020-12-16

**Authors:** Afeez Hazzan

**Affiliations:** State University of New York at Brockport, Hilton, New York, United States

## Abstract

Dementia is one of the most rapidly growing diseases in the United States. In 2018, the direct costs to American society of caring for older people with dementia was approximately $277 billion. Primary informal caregivers are mainly responsible for the care of older people with dementia including Alzheimer’s disease. Caregivers perform a myriad of duties ranging from shopping for their loved ones’ groceries, helping with medications, and managing finances. The caregiving role becomes more demanding as the disease progresses over time, and studies have shown that the quality-of-life (QoL) experienced by caregivers of older adults who have dementia is lower than the QoL of caregivers for older people who do not have dementia. To the best of our knowledge, there has been no research conducted to investigate whether lower caregiver QoL affects the level or quality of care that caregivers provide to persons with dementia. In the current study, we interviewed family caregivers living in Rochester, New York to inquire about their quality of life and the care provided to older people living with dementia. Further, caregivers completed the 36-item Short Form Health Survey (SF-36) as well as a draft questionnaire for measuring the quality of care provided to older people living with dementia. Both quantitative and qualitative findings from this study reveals important relationships between family caregiver QoL and the care provided, including the impact of social support and financial well-being. The study findings could have significant impact, particularly for the provision of much needed support for family caregivers.

